# Hyperinsulinemic Hypoglycemia in a Patient With a Mutation in the Insulin Receptor

**DOI:** 10.1210/jcemcr/luae221

**Published:** 2024-12-05

**Authors:** Marcus Imamovic, Mattias Vågberg, Kristina Cederquist, Per Dahlqvist

**Affiliations:** Department of Medicine, University Hospital of Umeå, 901 85 Umeå, Sweden; Department of Public Health and Clinical Medicine, Umeå University, 901 87 Umeå, Sweden; Department of Clinical Genetics, University Hospital of Umeå, 901 85 Umeå, Sweden; Department of Medical Biosciences, Umeå University, 901 87 Umeå, Sweden; Department of Clinical Genetics, University Hospital of Umeå, 901 85 Umeå, Sweden; Department of Medical Biosciences, Umeå University, 901 87 Umeå, Sweden; Department of Medicine, University Hospital of Umeå, 901 85 Umeå, Sweden; Department of Public Health and Clinical Medicine, Umeå University, 901 87 Umeå, Sweden

**Keywords:** INSR, insulin receptor gene, hyperinsulinemia, hypoglycemia, insulin-to-c-peptide ratio

## Abstract

Hyperinsulinemic hypoglycemias resulting from variants in the insulin receptor (*INSR*) gene are rare but clinically important disorders. We present a male patient in his 30s, experiencing recurrent postprandial hypoglycemic events. Endocrine evaluation revealed an elevated insulin-to-C-peptide ratio. A hypoglycemia gene panel, using next-generation sequencing, identified a heterozygous nonsense variant in the *INSR* gene (NM_000208.4) c.3079C > T, p.(Arg1027*). Initial treatment with diazoxide reduced hypoglycemic symptoms and led to weight loss and decreased hemoglobin A1c due to reduced compensatory carbohydrate intake. However, limiting side effects on diazoxide prompted a treatment switch to lanreotide with maintained absence of hypoglycemic events. This case highlights the importance of considering variants in the *INSR* gene as a differential diagnosis in hyperinsulinemic hypoglycemia cases, even in adults.

## Introduction

Although there is no universally accepted definition of hypoglycemia, it is often defined as plasma glucose level below 70 mg/dL (3.9 mmol/L) with concurrent adrenergic symptoms that include, but are not limited to, shakiness, irritability, tachycardia, sweating, and hunger. When plasma glucose level falls below 54 mg/dL (3.0 mmol/L), neuroglycopenic symptoms such as confusion, dizziness, behavioral changes, diplopia, seizures, and somnolence may occur [1]. Hypoglycemia in nondiabetic adults is uncommon. Endogenous hyperinsulinemic hypoglycemia is a condition characterized by dysregulated insulin secretion and/or clearance, leading to recurrent episodes of hypoglycemia [2]. The etiologies range from inborn errors of metabolism to acquired conditions including insulinoma and postbariatric surgery. Gene variants in the insulin receptor gene (*INSR*) represent a rare yet clinically relevant cause of hyperinsulinemic hypoglycemia. The insulin receptor plays a pivotal role in mediating the biological effects of insulin [3]. It is a transmembrane glycoprotein primarily expressed in insulin-responsive tissues such as skeletal muscle, adipose tissue, and liver. Activation of the insulin receptor initiates a cascade of intracellular signaling events that regulate glucose uptake, glycogen synthesis, lipid metabolism, and cell growth. Variants in the *INSR* gene can impair normal insulin receptor function and result in insulin resistance of varying severity. However, there are also a few reported cases of variants in the *INSR* gene associated with hyperinsulinemic hypoglycemia [4-8].

Our patient was diagnosed in adulthood with hyperinsulinemic hypoglycemia following a history of hypoglycemic symptoms since childhood.

## Case Presentation

A 33-year-old male patient was referred to the endocrine outpatient clinic because of suspected hypoglycemic events. Originally from southwestern Europe, he relocated to Sweden 9 years prior to the first visit. He had a history of allergic rhinitis from pollen, asthma, and diarrhea-predominant irritable bowel syndrome (IBS-D). His medication list included amitriptyline 20 mg at bedtime, loratadine 10 mg as needed, loperamide 2 mg as needed, psyllium daily, budesonide inhalation 100 μg twice per day, and terbutaline 200 μg inhalation as needed. He was not taking any supplements. He presented to his general practitioner with a history of recurrent postprandial symptoms of dizziness, diaphoresis, pallor, and fatigue several times weekly. He had acquired a standard blood glucose meter and observed that his symptoms coincided with capillary blood glucose levels between 45 and 49 mg/dL (2.5-2.7 mmol/L), with occasional levels even lower alongside presyncope. His symptoms typically started 2 to 4 hours postprandially, and never during fasting. The symptoms also presented during physical exercise and occasionally even when walking his dog. His symptoms were relieved upon consuming carbohydrates, which was paralleled with normalized capillary glucose levels, thereby meeting the criteria of the Whipple triad [2].

At presentation, he had no signs of acanthosis nigricans. His weight was 198 lb (90 kg) with a body mass index (BMI) of 33 kg/m^2^. A thorough medical history revealed recurrent similar events since childhood, initially a few times a year, gradually increasing to once or twice per month during adolescence. The frequency of the symptoms had increased over the past 5 years and was now significantly impacting his quality of life. Notably, his father, paternal uncle, and cousin experienced similar symptoms.

## Diagnostic Assessment

**Figure 1.**
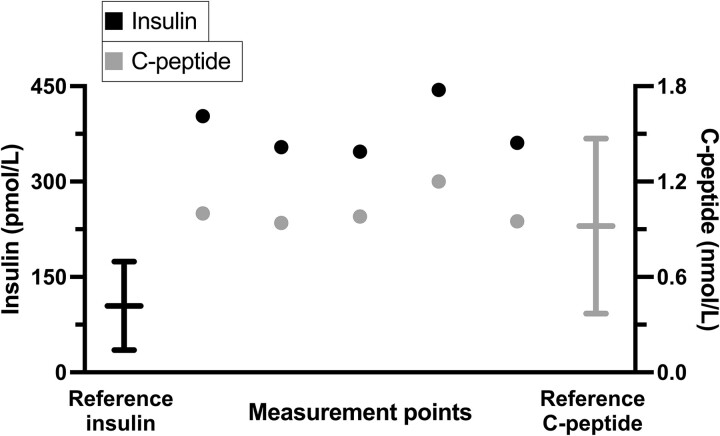
Results from measured fasting insulin and C-peptide levels at random dates and time points during the diagnostic assessment. Black whiskers to the left indicate the reference range for insulin. Gray whiskers to the right indicate the reference range for C-peptide. Note Système International (SI) units.

The diagnostic process was initiated with repeated fasting venous blood samples showing plasma glucose levels between 85 and 94 mg/dL (4.7-5.2 mmol/L) (reference range: 76-114 mg/dL; 4.2-6.3 mmol/L) with simultaneous normal plasma C-peptide levels of 2.8 to 3.0 ng/mL (.94-1.00 nmol/L) (reference range: 1.1-4.4 ng/mL; .37-1.47 nmol/L) but notably increased plasma insulin levels at 50 to 58 mIU/L (347-403 pmol/L) (reference range: 2.6-24.9 mIU/L; 35-174 pmol/L) and plasma proinsulin 23 to 33 pmol/L (reference range: 3.3-28 pmol/L) (Fig. 1). Hence, the insulin-to-C-peptide molar ratio was increased at .35 to .40 (normally <.1) [4]. Morning plasma cortisol was 12.1 µg/dL (335 nmol/L), serum IGF-1 was at 153 ng/mL (20.0 nmol/L) (reference range 73-246 ng/mL; 9.5-32.1 nmol/L) and ß-hydroxybutyrate was undetectable. Considering the history of IBS-D, laboratory testing for possible neuroendocrine tumor was conducted and results were unremarkable (Table 1).

**Table 1.** Serum results for assessment of neuroendocrine tumor

Measurand	Result	Reference range
Chromogranin A	25 ng/mL (.7 nmol/L)	<108 ng/mL (<3 nmol/L)
Pancreatic polypeptide	<84 pg/mL (<20 pmol/L)	<418 pg/mL (<100 pmol/L)
Gastrin	<10 pg/mL (<5 pmol/L)	<115 pg/mL (<55 pmol/L)
Glucagon	24 pg/mL (6.9 pmol/L)	<63 pg/mL (<18 pmol/L)
Vasoactive intestinal peptide	<25 pg/mL (<7.5 pmol/L)	<68 pg/mL (<20 pmol/L)
Calcitonin	.7 pg/mL (.2 pmol/L)	<9.6 pg/mL (<2.8 pmol/L)

Values are presented as conventional units (SI units).

He was admitted to the medical ward for a 72-hour fasting test. After a carbohydrate-rich breakfast, fasting started at 10 Am and ended prematurely after 49 hours because of intolerable tremor and diaphoresis, promptly relieved after intake of carbohydrates. At termination of fasting, his plasma glucose level was 52 mg/dL (2.9 mmol/L) and plasma insulin level was 9.2 mIU/L (64 pmol/L). The insulin-to-C-peptide molar ratio decreased after 24 hours of fasting but was still notably elevated at .21 (Table 2).

**Table 2.** Results of the 72-hour fasting test

Measurand	Time from test start						Reference range
	0 hours	12 hours	24 hours	36 hours	48 hours	49 hours	
Glucose	121 mg/dL (6.7 mmol/L)	**72 mg/dL** **(4.0 mmol/L)**	**72 mg/dL** **(4.0 mmol/L)**	**58 mg/dL** **(3.2 mmol/L)**	**56 mg/dL** **(3.1 mmol/L)**	**52 mg/dL** **(2.9 mmol/L)**	76-114 mg/dL (4.2-6.3 mmol/L)
Insulin	**348 mIU/L** **(2417 pmol/L)**	23 mIU/L (160 pmol/L)	13 mIU/L (90 pmol/L)	7.0 mIU/L (49 pmol/L)	6.2 mIU/L (43 pmol/L)	9.2 mIU/L (64 pmol/L)	2.6-24.9 mIU/L (35-174 pmol/L)
C-peptide			1.3 ng/mL (.42 nmol/L)				1.1-4.4 ng/mL (.37-1.47 nmol/L)
Insulin-to-C-peptide molar ratio			**.21**				<.10
Proinsulin			(7.4 pmol/L)				(3.3-28 pmol/L)
ß-hydroxybutyrate			**(.6 mmol/L)**				(<.3 mmol/L)

Values above or below the reference range are shown in bold font. Values are presented as conventional units (SI units).

Genetic investigation with a hypoglycemia gene panel using next-generation sequencing identified a heterozygous *INSR* nonsense variant (NM_000208.4) c.3079C > T, p.(Arg1027*). The variant was considered as pathogenic according to current American College of Medical Genetics criteria [9]. It has previously been described in heterozygous form associated with mild insulin resistance and has been shown to result in decreased mRNA levels [10, 11].

Our patient was provided with genetic counseling, including written and oral information about his genetic results. As the patient's relatives were residing outside of Sweden, further family investigation was not possible at our center. However, the patient was informed of the possibility for his relatives to pursue genetic counseling and investigation in their country of residence. The patient contacted his relatives, but none opted to undergo genetic testing. He has no biological children.

## Treatment

Our patient returned for follow-up 5 months later at the endocrine outpatient clinic and was now experiencing almost daily symptoms. His medications were reviewed again. He had initiated and discontinued sertraline 50 mg every day and mirtazapine 15 mg every day during the year of diagnostic assessment due to a depressive episode. He had discontinued his inhalations and started topical lidocaine ointment due to an anal fissure. No apparent triggers for increased hypoglycemic events were identified. He had tried several different diets, on his own initiative, without improvement. He had started compensating for the hypoglycemic episodes by eating excessively with subsequent weight gain and was now weighing 204 to 208 lb (92.5-94.5 kg) and BMI 34 to 35 kg/m^2^. He became extremely fatigued by the hypoglycemic events and was seeking a treatment option.

He was started on diazoxide 25 mg in the morning and received a continuous glucose monitor. Two weeks later, he reported fewer hypoglycemic events, with remaining episodes generally occurring in the evenings. Glucose monitoring indicated daytime levels ranging from 79 to 139 mg/dL (4.4-7.7 mmol/L) with a drop to 66 to 68 mg/dL (3.7-3.8 mmol/L) around 6 Pm, accompanied by reported symptoms. He was experiencing some side effects from diazoxide, mainly tachycardia, which restricted his ability to exercise. To further alleviate his hypoglycemic symptoms, the dose was increased by an additional 25 mg in the afternoon. He reported an initial paradoxical increase in hypoglycemic events for the first week, followed by no episodes the following week. The tachycardia persisted, prompting him to switch his physical exercise from cardio to weightlifting.

## Outcome and Follow-up

He was followed with regular laboratory assessments. During the first 6 months of treatment, glucose monitoring showed almost no glucose level below 70 mg/dL (3.9 mmol/L) and he reported very few hypoglycemic symptoms. His IBS-D symptoms seemed to improve, and he was able to discontinue the daily psyllium use. Notably, he also lost weight (−10 kg) and his hemoglobin A1c decreased from 5.9% to 5.4% (from 41 to 36 mmol/mol) (reference range: 4.6%-6.0%; 27-42 mmol/mol). After 6 months, he was still experiencing constraining tachycardia, especially during exercise. He was prescribed metoprolol 25 to 50 mg, which provided some relief. A 24-hour Holter monitoring showed sinus tachycardia 11% of the registered time.

Ten months after initiating diazoxide, he had a short period where he started experiencing increased episodes of nocturnal hypoglycemia with glucose levels as low as 49 mg/dL (2.7 mmol/L). He temporarily added another 25 mg of diazoxide in the evening. The nocturnal hypoglycemia immediately ceased and a few days later he was able to return to his previous dose.

After consulting Professor Kurt Højlund, University of Southern Denmark, Odense University Hospital, who was managing a Danish family with a variant in the *INSR* gene causing similar postprandial hypoglycemic episodes, the treatment was switched from diazoxide to lanreotide 60 mg every 4 weeks [4]. The side effects quickly subsided with maintained euglycemia. Our patient was able to start exercising cardio again and resume an ordinary lifestyle.

During the first year of hypoglycemic treatment, his weight continued to gradually decrease. At the latest follow-up, 17 months after initiation of diazoxide, bodyweight was 141 lb (64 kg) and BMI 24 kg/m^2^.

## Discussion

We present a case of hyperinsulinemic hypoglycemia presenting in adulthood, in a patient carrying a heterozygous variant in the *INSR* gene, which results in a premature stop codon and reduced mRNA levels, which is expected to lead to a decrease of available functioning insulin receptors. The variant has previously been described to cause insulin resistance in heterozygous form, which is rather the opposite of our patient's phenotype of hypoglycemic events. However, there is support for a role for the insulin receptor, not only in insulin signaling, but also in hepatic insulin clearance [3, 12]. This implies that an *INSR* gene variant leading to deficient, or functionally impaired, insulin receptors could potentially alter both peripheral insulin signaling and hepatic insulin clearance. Depending on the relative extents of these 2 effects, a variant in the *INSR* gene could possibly result in decreased insulin clearance, leading to increased levels of endogenous circulating insulin postprandially and thereby be causative of hypoglycemia. In our case, functional studies of the *INSR* nonsense variant would be required to ascertain this hypothesis, but its plausibility is underlined by previous reports of missense variants in the *INSR* gene being associated with hyperinsulinemic hypoglycemia [4-8]. Among these, the variant p.(Arg1174Gln) has been reported with a phenotype similar to our patient's phenotype [4]. In this family described by Højlund et al, the phenotype of hyperinsulinemic hypoglycemia was observed in association with an increased insulin-to-C-peptide molar ratio >.1, congruent with the consistently elevated insulin-to-C-peptide molar ratio of .35 to .40 seen in our patient (Fig. 1). Previous reports of patients with variants in the *INSR* gene and similar phenotypes have included oral glucose tolerance testing, showing excessive increases in plasma insulin levels [4-8]. Although our patient never underwent a conventional 75-g oral glucose tolerance test, we did see similar excessive insulin levels directly after the breakfast preceding the 72-hour fasting test with a plasma insulin level at 348 mIU/L (2417 pmol/L) (Table 2).

The 72-hour fasting test ended prematurely because of hypoglycemia, and the plasma insulin levels simultaneously increased slightly from 6.2 mIU/L (43 pmol/L) to 9.2 mIU/L (64 pmol/L), proposing a possible insulinoma. However, our patient had no clinical signs indicative of this, with symptoms since childhood, no history of hypoglycemia during fasting, no significantly low blood glucose levels (<2.5 mmol/L) during the test, and no impressively increased insulin-to-glucose ratio. We therefore considered insulinoma to be unlikely. The median biological coefficient of variation of plasma insulin is approximately 25% and we considered this to be the most probable cause of the slight increase [13]. We noted our patient was taking a once-daily low dose of amitriptyline to alleviate symptoms of IBS-D. Tricyclic antidepressants have previously been described in 2 cases in a case report to cause hypoglycemia in the setting of concomitant sulfonylurea use [14]. However, the patients in these cases were taking higher doses of tricyclic antidepressants and, looking retrospectively, we could not see any temporal association between our patient's long-term fixed dose of 20 mg at bedtime and the gradual increase in hypoglycemic events over many years. Consequently, we consider a link between patient's use of amitriptyline use and the hypoglycemic events to be unlikely.

Previously described cases have presented with a family history with a variety of clinical manifestations, spanning from hypoglycemia to insulin resistance, the latter more commonly found in the elderly [4, 5, 7]. For our patient, family studies were not possible as none of the patient's family members chose to pursue genetic testing. Because several other family members have been described to have experienced similar symptoms, family studies would have been valuable to further investigate the causality of the detected variant in the *INSR* gene. Considering the phenotypical spectrum of *INSR* gene variants and the consistently increased insulin levels, our patient will be closely monitored for the potential development of type 2 diabetes mellitus.

## Learning Points

- Variants in the *INSR* gene are rare yet clinically relevant causes of hyperinsulinemic hypoglycemia.
- Variants in the *INSR* gene should be considered as a differential diagnosis in patients presenting with postprandial hypoglycemia, even in adults.
- Continuous glucose monitoring is a valuable tool in the treatment and management of hypoglycemia.

## Data Availability

Original data generated and analyzed during this study are included in this published article.

## Abbreviations

BMI: body mass index
IBS-D: diarrhea-predominant irritable bowel syndrome
